# HLA-G in Allergy: Does It Play an Immunoregulatory Role?

**DOI:** 10.3389/fimmu.2021.789684

**Published:** 2022-01-10

**Authors:** Simone Negrini, Paola Contini, Giuseppe Murdaca, Francesco Puppo

**Affiliations:** Department of Internal Medicine, University of Genoa, Genoa, Italy

**Keywords:** HLA-G, soluble HLA-G, allergy, allergic rhinitis, allergic asthma

## Abstract

Allergy is an inflammatory process determined by a cascade of immune events characterized by T-helper 2 lymphocytes polarization leading to interleukin-4 upregulation, IgE secretion, and mast cell and eosinophil activation. HLA-G molecules, both in membrane-bound and in soluble forms, are known to play a key immunoregulatory role and their involvement in allergic diseases is supported by increasing literature data. HLA-G expression and secretion is specifically induced in peripheral blood mononuclear cells of allergic patients after *in vitro* incubation with the causal allergen. Elevated levels of soluble HLA-G molecules are detected in serum of patients with allergic rhinitis correlating with allergen-specific IgE levels, clinical severity, drug consumption and response to allergen-specific immunotherapy. HLA-G genetic polymorphisms confer susceptibility to allergic asthma development and high levels of soluble HLA-G molecules are found in plasma and bronchoalveolar lavage fluid of patients with allergic asthma correlating with allergen-specific IgE levels. Interestingly, allergic pregnant women have lower plasma sHLA-G levels than non-allergic women during the 3^rd^ trimester of pregnancy and at delivery. Finally, in allergic patients with atopic dermatitis HLA-G molecules are expressed by T cells, monocytes-macrophages and Langerhans cells infiltrating the dermis. Although at present is difficult to completely define the role of HLA-G molecules in allergic diseases, it may be suggested that they are specifically expressed and secreted by immune cells during the allergic reaction in an attempt to suppress allergic inflammation.

## Introduction

### HLA-G

Human leukocyte antigen-G (HLA-G) is an HLA class Ib antigen characterized by a restricted tissue expression, low polymorphism and 7 isoforms (HLA-G1 to HLA-G7) generated by alternative splicing of the primary HLA-G transcript (1, 2). Four of them, HLA-G1, -G2, -G3 and -G4, are bound to the cell surface, while the remaining three, HLA-G5, -G6 and -G7, are detectable in soluble form (sHLA-G) (1). HLA-G, both membrane-bound and soluble form, exerts several immune-modulatory effects. In fact, it inhibits CD4+ T cells allogeneic proliferation (3), natural killer (NK) and CD8+ T cells cytotoxicity (4), dendritic cells (DC) maturation (5), and B cells activation (6). In addition, soluble HLA-G molecules (sHLA-G) trigger apoptosis in antigen specific CD8+ T lymphocytes (4, 7, 8).

Furthermore, HLA-G may induce immune tolerance leading to the development of tolerogenic DC with induction of anergic and immunosuppressive T cells promoting the expansion of CD4+ CD25+ FoxP3+ T regulatory lymphocytes (Tregs) and triggering the differentiation of CD4+ T-cells in suppressor cells (9). HLA-G seems also to be involved in the tuning of immune responses, as incubation of peripheral blood mononuclear cells (PBMC) with HLA-G-expressing cells favors a shift towards a Th-2 cytokine profile, whereas incubation with soluble HLA-G molecules may have a counterbalancing effect by creating an anti-inflammatory environment due to the release of interleukin (IL)-10 (10, 11). While originally described as restricted in its constitutive tissue expression (12–16), HLA-G expression can be induced in several pathologic conditions (17–24). Of note, cytokines such as interferon (IFN)-γ and IL-10 trigger the expression of HLA-G by PBMC. Particularly, IL-10 enhances HLA-G expression and down-regulates classical HLA class I and class II antigens expression on monocytes, thus regulating NK cells and T lymphocyte responses (25, 26).

Recently, a novel subset of thymus-derived T lymphocytes expressing HLA-G have been described as distinct population of Tregs (27). HLA-G+ Tregs can be differentiated from classical CD4+ Tregs because of the phenotype lacking Forkhead Box P3 (FoxP3), CD39 and CD25 expression (27) and since mediate their immunomodulatory functions through cell-to-cell contact independent mechanisms (28) whereas classical CD4+CD25+FoxP3+ Tregs act mainly *via* cell-to-cell mechanisms (29). HLA-G-expressing Tregs exert their regulatory activity through various tolerogenic soluble molecules such as sHLA-G5, IL-10, IL-35 and transforming growth factor (TGF)-β (30, 31). Besides thymus-derived HLA-G+ Tregs, normal CD4+ and CD8+ T cells may acquire the HLA-G1 molecule from antigen presenting cells (APCs) through trogocytosis thus modulating their function from effectors to regulatory cells capable to inhibit alloproliferative responses (32). Interestingly, the acquisition of HLA-G *via* trogocytosis mechanism has also been reported for NK cells and monocytes (33, 34). A non-cytolytic subset of NK cells expressing HLA-G (NK-ireg) can be generated *in vitro* from peripheral blood CD34+ hematopoietic progenitors. NK-ireg cells display a mature NK cell phenotype, release suppressive molecules (sHLA-G, IL-10, IL-21) and through these factors down-modulate DCs activity and NK cells cytotoxicity (35).

HLA-G+ immune cells are present in the peripheral blood of healthy subjects where they probably contribute to maintain immune tolerance. Conversely, increased percentages of circulating and tissue-infiltrating HLA-G+ immune cells (e.g., T and NK cells, monocytes, DCs, mast cells) can be observed in different pathological situations such as infections, cancers, transplants and autoimmune disorders suggesting a potential role for these cells in the pathogenesis of diseases in which immune system is strictly implicated (1, 2, 24, 36–38). Based on these findings, it has been proposed that HLA-G should be qualified as an ‘immune checkpoint’ molecule (39).

### Allergy

Allergic diseases are characterized by an IgE mediated antibody response to an environmental allergen. Both genetic and environmental factors contribute to the development of allergic disease. Exposure of a genetically predisposed individual to allergen results in uptake of the allergen by APC followed by intracellular digestion of the allergen into peptide fragments and display of the peptide fragments by HLA on the APC membrane. Allergen-specific T helper cells type 2 (Th2) interact with the APC and secrete cytokines like interleukin (IL)-4, IL-5 and IL-13 which induce mast cell, basophil and eosinophil proliferation and IgE production by B cells. In addition, it is now appreciated that other T cell types, such as Th17 and Th9 may be involved in allergy development (40). Cross-linking of FcϵRI through allergen-IgE binding sensitizes mast cells and basophils to release biologically active mediators including histamine, serotonin, proteoglycans, tryptase, leukotrienes and prostaglandins causing the allergic reaction and tissue inflammation (40). Recent studies have identified a new type of Th cells localized in B-cell follicles in the secondary lymphoid organs, named follicular helper T (Tfh) cells, that produce IL-4 and IL-13 and regulate antibody isotype switching required for IgE production (41). Furthermore, a subset of Tregs has been identified within lymphoid follicles that counteract Tfh cells and suppress IgE production thus preventing allergic responses (41). Therefore, a tilted balance in the Tfh/Tregs axis may represent an essential feature of allergic diseases. From a clinical perspective, allergic diseases comprise allergic rhinitis, asthma, conjunctivitis and dermatitis, and food allergy.

## HLA-G and Allergic Rhinitis

Allergic rhinitis is sustained by mucosal IgE-dependent inflammation characterized by mast cell and eosinophil activation.

Our group investigated sHLA-G serum levels in adult allergic rhinitis patients allergic to seasonal and perennial allergens. Serum sHLA-G levels are significantly higher in allergic patients as compared to healthy controls and strongly correlate with allergen-specific IgE levels as well as with rhinitis clinical severity and anti-allergic drug consumption (**Table 1**). Of interest, serum sHLA-G levels are higher in patients with seasonal allergy than in those with perennial allergy (42–46, 51) Moreover, sHLA-G levels significantly decrease 3 months after the end of allergen-specific immunotherapy and correlate with the increased production of IFN-γ by peripheral blood mononuclear cells suggesting a successful shift from Th2 to Th1 immune response (52, 53).

**Table 1:** sHLA-G plasma levels in Allergic Rhinitis and Asthma.

	Patients	Controls	*Ref. n.*
Allergic rhinitis	35.86*	12.79	(42)
	42.80	9.80	(43)
	35.38	9.76	(44)
	24.68	7.03	(45)
	46.36	6.75	(46)
			
Asthma (Children)	67.9**	n.a.	(47)
	52	42	(48)
	179.3	35.2	(49)
			
Bronchoalveolar lavage (adults)	6.8	1.6	(50)

*ng/mL; **U/mL.

These data agree with those recently published by another research group revealing that allergic rhinitis patients have significantly higher serum sHLA-G levels than normal subjects and that there is a highly significant and positive correlation between sHLA-G and specific IgE levels (54). Finally, elevated serum sHLA-G amounts have been also found in children with allergic diseases (49, 55) (**Table 1**).

## HLA-G and Asthma

Allergic asthma is characterized by persistent airway inflammation, structural remodelling and bronchial hyperresponsiveness in lower airways driven by Th2 lymphocytes activation and IL-4, IL-5 and IL-13 release.

Genetic factors play a central role in asthma pathogenesis and over 100 genes have been implicated in asthma susceptibility. The potential involvement of HLA-G in asthma development has been suggested by pivotal studies indicating a linkage between asthma and chromosome 6p21 (56, 57). Particularly, HLA-G polymorphisms may confer susceptibility to airway hyperresponsiveness and asthma development. The G/G genotype at SNP -964 G/A in the promoter region is associated with asthma in the offspring of mothers with asthma or bronchial hyperresponsiveness, while the A/A genotype is associated with asthma in the offspring of asthma-free and hyperresponsiveness-free mothers (57).

In following years, a genome-wide association study (GWAS) performed in 6819 participants from the Framingham Heart Study identified potential susceptibility loci in the HLA-G gene regions as risk factors for IgE dysregulation and atopy (58).

Further studies analyzed the potential interaction among maternal asthma, microRNA regulation of soluble HLA-G in the airway and offspring subsequent risk for asthma. Variants in the HLA-G 3′ UTR including SNP +3142 C/G (rs1063320) that disrupts a target site for the microRNA (miR)-152 family were evaluated. Results indicated that +3142 genotypes were associated with elevated miR-148b and sHLA-G concentrations in BAL fluid among asthmatic subjects with an asthmatic mother but not among those with a non-asthmatic mother. These results are consistent with +3142 allele-specific targeting of HLA-G by the miR-152 family and support the hypothesis that miRNA regulation of sHLA-G in the airway is influenced by both the asthma status of the subject’s mother and the subject’s genotype (59, 60).

More recently, HLA-G haplotypes were characterized by next generation sequencing from position −1983 to +3447 and sHLA-G serum levels were quantified both in a cohort of 330 healthy subjects and in 580 asthmatic patients from a French multicenter cohort. HLA-G haplotypes displayed statistically significant differential distribution between healthy subjects and asthmatic patients and a significant association with eosinophil count as well as with history of near-fatal asthma and asthma exacerbations. By contrast, no association was found between sHLA-G serum level and genetic data suggesting the hypothesis that sHLA-G is not overexpressed as a systemic immune response to control local inflammation (61).

The potential role of HLA-G in asthma has been also reported in a Brazilian study evaluating the HLA-G untranslated region (3′UTR) in 115 asthmatic patients stratified according to disease severity (mild, moderate, and severe) and in 116 healthy individuals. The +3010C and +3142G alleles were overrepresented in mild asthma patients when compared to controls and the +3010G and +3142C alleles were overrepresented in severe asthma patients in comparison to patients with mild asthma. These results suggest that HLA-G 3′UTR segment variation sites were differentially associated according to asthma severity (62).

A role for HLA-G in asthma pathogenesis is further suggested by the demonstration of sHLA-G molecule expression in the airway epithelium and of increased levels of sHLA-G in plasma and bronchoalveolar lavage (BAL) fluid of children with atopic asthma (47–49) (**Table 1**). However, no significant association was observed between plasma sHLA-G, total IgE and allergen specific IgE levels. Moreover, sHLA-G levels were not significantly related to HLA-G 14-bp insertion/deletion polymorphism. Other studies indicated that, among subjects with asthma, BAL sHLA-G concentrations were inversely correlated with markers of inflammation in the airway. In particular, sHLA-G concentrations were highest in subjects with low BAL eosinophils, low fractional exhaled nitric oxide (FENO), a marker of airways inflammation, and low serum IgE suggesting that sHLA-G concentrations were highest in patients with low inflammatory endotype of asthma and best pulmonary function (50, 63) (**Table 1**). Interestingly, bronchial epithelial cells from patients with mild and severe asthma display impaired mRNA expression of HLA-G1, -G4, and -G5 functional isoforms and HLA-G expression is not affected by IL-13 supporting the hypothesis that an impaired expression of HLA-G isoforms in asthmatic patients could contribute to the loss of inflammation control and epithelium structural remodeling (64).

Of note, it has been reported that in infants with asthma sHLA-G plasma levels were significantly higher in subjects with persistent wheezing compared with subjects with transient wheezing. However, there was no significant difference in peripheral blood eosinophil count and total IgE level between the two groups. These results may suggest that the increased sHLA-G levels in infants with persistent wheeze may be able to be used to distinguish persistent from transient wheeze (47).

The potential role of pregnancy and labor on plasma sHLA-G levels was evaluated in allergic and non-allergic women (65). Plasma samples were obtained during the 3^rd^ trimester of pregnancy, at delivery and at a non-pregnant state 2 years post-partum. Levels of the sHLA-G1 isoform in plasma significantly increased during labor compared to levels detected during the 3rd trimester of pregnancy and two years after delivery. However, allergic women had lower plasma sHLA-G levels than non-allergic women during the 3^rd^ trimester of pregnancy and at delivery. Interestingly, no significant differences were found in samples obtained 2 years after pregnancy. Finally, spontaneous production of sHLA-G by PBMCs resulted significantly higher in patients with isocyanate-induced asthma than in other groups of asthmatic patients (66).

HLA-G binding of KIR2DL4 (CD158d) receptor on NK cells induces the secretion IFN-γ, a cytokine critical for the generation of tolerogenic DC. As consequence it might be predicted that individuals with a functionally defective allele of KIR2DL4 would not be able to secrete IFNγ and might therefore be prone to Th2-biased immune responses and produce fewer tolerogenic DC. KIR2DL4 genotypes were analyzed in 2 cohorts of children at high risk for atopic disease and asthma. However, there was no significant relationship between KIR2DL4 genotype and the prevalence of atopy and asthma (67).

It has been suggested that infections may play a role in the pathophysiology of allergic diseases, in particular asthma. The relationship among allergy, infections and HLA-G is an intriguing question, however no data are currently available on this topic.

## HLA-G and Atopic Dermatitis

Atopic dermatitis (AD) is a chronic disease usually beginning in childhood. AD is characterized by increased production of IL-4, IL-13 and IgE. In AD biopsies, HLA-G positive cells were always found in the papillary and, less frequently, in the reticular dermis. HLA-G was expressed mainly by infiltrating T cells but also, to a lesser extent and less frequently, by monocytes-macrophages or Langerhans cells (20). It is noteworthy that topical administration of purified recombinant HLA-G1 ameliorate the AD-like skin lesions in the mice. In addition, serum levels of IgE, IL-13, and IL-17A are significantly reduced in HLA-G1-treated mice. Taken together, these observations suggest a potential role for recombinant HLA-G as novel therapeutic strategy for AD and other chronic inflammatory skin disorders (68).

## *In Vitro* Data

The *in vitro* expression and release of HLA-G molecules by PBMC after incubation with both allergenic and non-allergenic stimuli was evaluated in allergic rhinitis patients HLA-G membrane expression was specifically induced by incubation with the causal allergen, but not by incubation with non-causal allergens or non-specific stimuli. Monocytes and to a lesser extent CD4+ T cells, particularly Th2 cells, expressed HLA-G after allergenic challenge whereas CD8+ T lymphocytes, B lymphocytes, NK cells and Tregs did not show any detectable HLA-G expression after incubation with allergens. The exposure to the causal allergen seems to be the main factor inducing HLA-G expression. In fact, patients allergic to mites, evaluated during winter, when the exposure to mite was still present, showed the more intense membrane HLA-G expression, whereas grass pollen allergic patients, who were evaluated far from the pollen season, showed a very low increase of HLA-G expression. The measurement of sHLA-G in culture supernatants confirmed that high amounts of sHLA-G molecules are found when the causal allergen is used as stimulus. Soluble molecules detected in culture supernatants mainly belong to the HLA-G5 isoform suggesting that they are actively secreted by immune cells after incubation with allergen (69, 70).

## Conclusions

**Figure 1:**
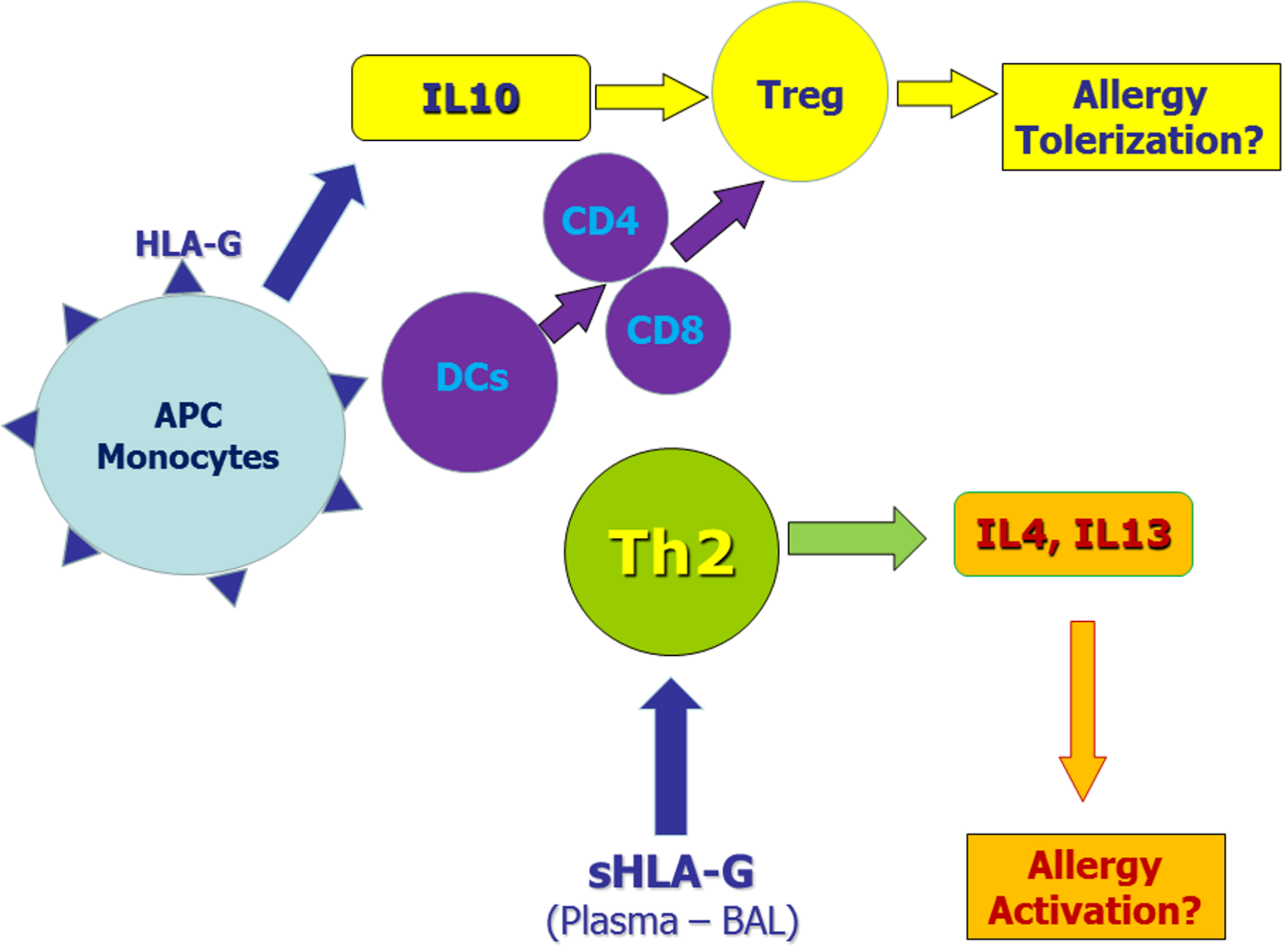
Potential role of membrane-bound and soluble HLA-G molecules in allergic diseases. Monocytes and antigen presenting cells (APC) expressing membrane-bound HLA-G molecules secrete IL-10 and induce tolerogenic dendritic cells. These mechanisms induce regulatory T cells (Tregs) that may exert tolerogenic effects on the allergic process. On the other hand, soluble HLA-G molecules in plasma and/or bronchoalveolar lavage (BAL) may facilitate Th2 polarization thus sustaining allergic responses.

HLA-G molecules have a complex immune regulatory role in transplantation, cancer, viral infections, chronic inflammatory diseases and pregnancy (**Table 2**). In general, HLA-G is a tolerance-inducing molecule by inducing Treg cells, but it is also a pro-inflammatory molecule stimulating Th2 responses (**Figure 1**). Allergic diseases are driven by a Th2-polarized inflammation and allergic patients display a defect in Treg cells which may be restored by specific immunotherapy. Taken together, the studies reported in this review suggest that: i) sHLA-G plasma levels are greater in atopic than in normal subjects and decrease after specific immunotherapy; ii) HLA-G is an asthma susceptibility gene; iii) HLA-G molecules are present in airway epithelium and BAL fluid of asthmatic subjects; iv) HLA-G is expressed and secreted by immune cells of atopic patients following *in vitro* allergenic challenge. At present, it remains unclear whether the presence of HLA-G is reactive in attempt to restore a proper balance in inflammatory cells and cytokines activated in allergic diseases or is a part of their pathogenesis by diverting the immune response towards a Th2 phenotype or by altering the presence and function of Treg cells. This latter hypothesis is supported by the finding that antigen presenting cells and monocytes expressing HLA-G molecules create a tolerogenic milieu enriched in IL-10 which, in turn, promotes Treg cells activity. In conclusion, it could be postulated that HLA-G molecules in allergy may be either compensatory or pathogenetic, but their precise mechanism of action is not yet completely known and needs further investigation.

**Table 2:** HLA-G in non-allergic diseases.

Disease	Analyzed data	Pathophysiologic relevance	Ref. n.
Chron’s disease	Polymorphisms	Increased disease susceptibility	(71)
	Elevated serum levels	Positive correlation with disease severity	
Rheumatoid arthritis	Polymorphisms	Increased disease susceptibility	(72)
	Elevated serum levels	Negative correlation with disease severity	
Systemic lupus erythematosus	Polymorphisms	Increased disease susceptibility	(73, 74)
	Elevated expression and serum levels	Positive correlation with disease severity	
Systemic sclerosis	Low serum levels	Negative correlation with disease severity	(75, 76)
	Elevated serum levels	No correlation with disease severity	
Multiple sclerosis	Polymorphisms	Increased disease susceptibility	(77, 78)
	Elevated dimer levels	Positive correlation with decreased inflammation	
Psoriasis	Polymorphisms	Positive correlation with treatment response	(79)
Toxoplasmosis	Elevated trophoblast release	Abnormal pregnancy	(80)
Malaria	Elevated cord blood levels	Low weight at birth and infection risk	(81)
*Helycobacter pylori* infection	Increased expression	Negative correlation with inflammation	(82)
HCV – HBV – HIV	Polymorphisms	Worse outcome and response to treatment	(83–85)
	Elevated serum levels		
Tumors (Gastrointestinal, kidney, breast, lung, melanoma)	Increased expression and serum levels	Increased metastasis and worse outcome	(86–92)

## Author Contributions

All authors equally contributed to the conception of ideas and design of this manuscript.

## Funding

The study was supported by Università degli Studi di Genova.

## Conflict of Interest

The authors declare that the research was conducted in the absence of any commercial or financial relationships that could be construed as a potential conflict of interest.

## Publisher’s Note

All claims expressed in this article are solely those of the authors and do not necessarily represent those of their affiliated organizations, or those of the publisher, the editors and the reviewers. Any product that may be evaluated in this article, or claim that may be made by its manufacturer, is not guaranteed or endorsed by the publisher.

## References

[1] Gonzalez A Rebmann V LeMaoult J Horn PA Carosella ED Alegre E. The Immunosuppressive Molecule HLA-G and Its Clinical Implications. Crit Rev Clin Lab Sci (2012) 49:63–84. doi: 10.3109/10408363.2012.677947 22537084

[2] LeMaoult J Yan WA. Editorial: The Biological and Clinical Aspects of HLA-G. Front Immunol (2021) 12:649344. doi: 10.3389/fimmu.2021.649344 33679813 PMC7933029

[3] Lila N Rouas-Freiss N Dausset J Carpentier A Carosella ED. Soluble HLA-G Protein Secreted by Allo-Specific CD4+ T Cells Suppresses the Allo-Proliferative Response: A CD4+ T Cell Regulatory Mechanism. Proc Natl Acad Sci USA (2001) 98:12150–5. doi: 10.1073/pnas.201407398 PMC59783 11572934

[4] Contini P Ghio M Poggi A Filaci G Indiveri F Ferrone S. Soluble HLA-A,-B,-C and -G Molecules Induce Apoptosis in T and NK CD8+ Cells and Inhibit Cytotoxic T Cell Activity Through CD8 Ligation. Eur J Immunol (2003) 33:125–34. doi: 10.1002/immu.200390015 12594841

[5] Le Friec G Laupeze B Fardel O Sebti Y Pangault C Guilloux V. Soluble HLA-G Inhibits Human Dendritic Cell-Triggered Allogeneic T-Cell Proliferation Without Altering Dendritic Differentiation and Maturation Processes. Hum Immunol (2003) 64:752–61. doi: 10.1016/s0198-8859(03)00091-0 12878353

[6] Naji A Menier C Morandi F Agaugue S Maki G Ferretti E. Binding of HLA-G to ITIM-Bearing Ig-Like Transcript 2 Receptor Suppresses B Cell Responses. J Immunol (2014) 192:1536–46. doi: 10.4049/jimmunol.1300438 24453251

[7] Contini P Ghio M Merlo A Poggi A Indiveri F Puppo F. Apoptosis of Antigen-Specific T Lymphocytes Upon the Engagement of CD8 by Soluble HLA Class I Molecules is Fas Ligand/Fas Mediated: Evidence for the Involvement of P56lck, Calcium Calmodulin Kinase II, and Calcium-Independent Protein Kinase C Signaling Pathways and for NF-kappaB and NF-AT Nuclear Translocation. J Immunol (2005) 175:7244–54. doi: 10.4049/jimmunol.175.11.7244 16301629

[8] Fournel S Aguerre-Girr M Huc X Lenfant F Alam A Toubert A. Cutting Edge: Soluble HLA-G1 Triggers CD95/CD95 Ligand-Mediated Apoptosis in Activated CD8+ Cells by Interacting With CD8. J Immunol (2000) 164:6100–4. doi: 10.4049/jimmunol.164.12.6100 10843658

[9] Ristich V Liang S Zhang W Wu J Horuzsko A. Tolerization of Dendritic Cells by HLA-G. Eur J Immunol (2005) 35:1133–42. doi: 10.1002/eji.200425741 15770701

[10] Kanai T Fujii T Unno N Yamashita T Hyodo H Miki A. Human Leukocyte Antigen-G-Expressing Cells Differently Modulate the Release of Cytokines From Mononuclear Cells Present in the Decidua Versus Peripheral Blood. Am J Reprod Immunol (2001) 45:94–9. doi: 10.1111/j.8755-8920.2001.450205.x 11216880

[11] Kanai T Fujii T Kozuma S Yamashita T Miki A Kikuchi A. Soluble HLA-G Influences the Release of Cytokines From Allogeneic Peripheral Blood Mononuclear Cells in Culture. Mol Hum Reprod (2001) 7:195–200. doi: 10.1093/molehr/7.2.195 11160846

[12] Kovats S Main EK Librach C Stubblebine M Fisher SJ DeMars R. A Class I Antigen, HLA-G, Expressed in Human Trophoblasts. Science (1990) 248:220–3. doi: 10.1126/science.2326636 2326636

[13] Mallet V Blaschitz A Crisa L Schmitt C Fournel S King A. HLA-G in the Human Thymus: A Subpopulation of Medullary Epithelial But Not CD83(+) Dendritic Cells Expresses HLA-G as a Membrane-Bound and Soluble Protein. Int Immunol (1999) 11:889–98. doi: 10.1093/intimm/11.6.889 10360962

[14] Le Discorde M Moreau P Sabatier P Legeais JM Carosella ED. Expression of HLA- G in Human Cornea, an Immune-Privileged Tissue. Hum Immunol (2003) 64:1039–44. doi: 10.1016/j.humimm.2003.08.346 14602233

[15] Cirulli V Zalatan J McMaster M Prinsen R Salomon DR Ricordi C. The Class I HLA Repertoire of Pancreatic Islets Comprises the Nonclassical Class Ib Antigen HLA-G. Diabetes (2006) 55:1214–22. doi: 10.2337/db05-0731 16644675

[16] Menier C Rabreau M Challier JC Le Discorde M Carosella ED Rouas-Freiss N. Erythroblasts Secrete the Nonclassical HLA-G Molecule From Primitive to Definitive Hematopoiesis. Blood (2004) 104:3153–60. doi: 10.1182/blood-2004-03-0809 15284117

[17] Rouas-Freiss N Moreau P Ferrone S Carosella ED. HLA-G Proteins in Cancer: Do They Provide Tumor Cells With an Escape Mechanism? Cancer Res (2005) 65:10139–44. doi: 10.1158/0008-5472.CAN-05-0097 16287995

[18] Lila N Carpentier A Amrein C Khalil-Daher I Dausset J Carosella ED. Implication of HLA-G Molecule in Heart-Graft Acceptance. Lancet (2000) 355:2138. doi: 10.1016/S0140-6736(00)02386-2 10902633

[19] Wiendl H Feger U Mittelbronn M Jack C Schreiner B Stadelmann C. Expression of the Immune-Tolerogenic Major Histocompatibility Molecule HLA-G in Multiple Sclerosis: Implications for CNS Immunity. Brain (2005) 128:2689–704. doi: 10.1093/brain/awh609 16123145

[20] Khosrotehrani K Le Danff C Reynaud-Mendel B Dubertret L Carosella ED Aractingi S. HLA-G Expression in Atopic Dermatitis. J Invest Dermatol (2001) 117:750–2. doi: 10.1046/j.0022-202x.2001.01487.x 11564188

[21] Aractingi S Briand N Le Danff C Viguier M Bachelez H Michel L. HLA-G and NK Receptor Are Expressed in Psoriatic Skin: A Possible Pathway for Regulating Infiltrating T Cells? Am J Pathol (2001) 159:71–7. doi: 10.1016/S0002-9440(10)61675-6 PMC1850403 11438456

[22] Lozano JM Gonzalez R Kindelan JM Rouas-Freiss N Caballos R Dausset J. Monocytes and T Lymphocytes in HIV-1- Positive Patients Express HLA-G Molecule. Aids (2002) 16:347–51. doi: 10.1097/00002030-200202150-00005 11834945

[23] Lafon M Prehaud C Megret F Lafage M Mouillot G Roa M. Modulation of HLA-G Expression in Human Neural Cells After Neurotropic Viral Infections. J Virol (2005) 79:15226–37. doi: 10.1128/JVI.79.24.15226-15237.2005 PMC1316015 16306594

[24] Contini P Murdaca G Puppo F Negrini S. HLA-G Expressing Immune Cells in Immune Mediated Diseases. Front Immunol (2020) 11:1613. doi: 10.3389/fimmu.2020.01613 32983083 PMC7484697

[25] Yang Y Chu W Geraghty DE Hun JS. Expression of HLA-G in Human Mononuclear Phagocytes and Selective Induction by IFN-Gamma. J Immunol (1996) 156:4224–31. 8666791

[26] Moreau P Adrian-Cabestre F Menier C Guiard V Gourand L Dausset J. IL-10 Selectively Induces HLA-G Expression in Human Trophoblasts and Monocytes. Int Immunol (1999) 11:803–11. doi: 10.1093/intimm/11.5.803 10330285

[27] Feger U Tolosa E Huang YH Waschbisch A Biedermann T Melms A. HLA-G Expression Defines a Novel Regulatory T-Cell Subset Present in Human Peripheral Blood and Sites of Inflammation. Blood (2007) 110:568–77. doi: 10.1182/blood-2006-11-057125 17371944

[28] Huang YW Zozulya AL Weidenfeller C Schwab N Wiendl H. T Cell Suppression by Naturally Occurring HLA-G-Expressing Regulatory CD4+ T Cells is IL-10-Dependent and Reversible. J Leukoc Biol (2009) 86:273–81. doi: 10.1189/jlb.1008649 19401389

[29] Zhao H Liao X Kang Y. Tregs: Where We Are and What Comes Next? Front Immunol (2017) 8:1578. doi: 10.3389/fimmu.2017.01578 29225597 PMC5705554

[30] Pankratz S Bittner S Herrmann AM Schuhmann MK Ruck T Sven G Meuth SG. Human CD4+ HLA-G+ Regulatory T Cells Are Potent Suppressors of Graft-Versus-Host Disease In Vivo. FASEB J (2014) 28:3435–45. doi: 10.1096/fj.14-251074 24744146

[31] Carosella ED Gregori S LeMaoult J. The Tolerogenic Interplay(s) Among HLA-G, Myeloid APCs, and Regulatory Cells. Blood (2011) 118:6499–505. doi: 10.1182/blood-2011-07-370742 21960588

[32] LeMaoult J Caumartin J Daouya M Favier B Le Rond S Gonzalez A. Immune Regulation by Pretenders: Cell-to-Cell Transfers of HLA-G Make Effector T Cells Actas Regulatory Cells. Blood (2007) 109:2040–8. doi: 10.1182/blood-2006-05-024547 17077329

[33] Caumartin J Favier B Daouya M Guillard C Moreau P Carosella ED. Trogocytosis-Based Generation of Suppressive NK Cells. EMBO J (2007) 26:1423–33. doi: 10.1038/sj.emboj.7601570 PMC1817622 17318190

[34] HoWangYin KY Alegre E Daouya M Favier B Carosella ED LeMaoult J. Different Functional Outcomes of Intercellular Membrane Transfers to Monocytes and T Cells. Cell Mol Life Sci (2010) 67:1133–45. doi: 10.1007/s00018-009-0239-4 PMC11115494 20238479

[35] Giuliani M Giron-Michel J Negrini S Vacca P Durali D Caignard A. Generation of a Novel Regulatory NK Cell Subset From Peripheral Blood CD34+ Progenitors Promoted by Membrane-Bound IL-15. PloS One (2008) 3:e2241. doi: 10.1371/journal.pone.0002241 18493613 PMC2376096

[36] Carosella ED HoWangYin KY Favier B LeMaoult J. HLA-G-Dependent Suppressor Cells: Diverse by Nature, Function, and Significance. Hum Immunol (2008) 69:700–7. doi: 10.1016/j.humimm.2008.08.280 18817832

[37] Lin A Yan WH. Intercellular Transfer of HLA-G: Its Potential in Cancer Immunology. Clin Transl Immunol (2019) 8:e1077. doi: 10.1002/cti2.1077 PMC6716982 31489189

[38] Lin A Yan WH. HLA-G as an Inhibitor of Immune Responses. Methods Mol Biol (2016) 1371:3–9. doi: 10.1007/978-1-4939-3139-2_1 26530791

[39] Carosella ED Rouas-Freiss N Tronik-Le Roux D Moreau P LeMaoult J. HLA-G: An Immune Checkpoint Molecule. Adv Immunol (2015) 127:33–144. doi: 10.1016/bs.ai.2015.04.001 26073983

[40] Oettgen H Broide DH. Introduction to Mechanisms of Allergic Disease. In: Allergy. Holgate ST Broide DH Martinez FD, eds. Edinburgh: Elsevier Saunders (2012). p. 1–32.

[41] Yao Y Chen CL Yu D Liu Z. Roles of Follicular Helper and Regulatory T Cells in Allergic Diseases and Allergen Immunotherapy. Allergy (2021) 76:456–70. doi: 10.1111/all.14639 33098663

[42] Ciprandi G Colombo BM Contini P Cagnati P Pistorio A Puppo F. Soluble HLA-G and HLA-A,-B,-C Serum Levels in Patients With Allergic Rhinitis. Allergy (2008) 63:1335–8. doi: 10.1111/j.1398-9995.2008.01741.x 18782112

[43] Ciprandi G Contini P Murdaca G Gallina AM Puppo F. Soluble HLA-G Molecules in Patients With Perennial Allergic Rhinitis. Int Arch Allergy Immunol (2009) 150:278–81. doi: 10.1159/000222680 19494525

[44] Ciprandi G Contini P Murdaca G DeAmici M Gallina AM Puppo F. Soluble Serum HLA-G and HLA-A, -B, -C Molecules in Patients With Seasonal Allergic Rhinitis Exposed to Pollens. Int Immunopharmacol (2009) 9:1058–62. doi: 10.1016/j.intimp.2009.04.014 19410660

[45] Ciprandi G Corsico A Pisati P. Serum-Soluble HLA-G is Associated With Specific IgE in Patients With Allergic Rhinitis and Asthma. Inflammation (2014) 37:1630–4. doi: 10.1007/s10753-014-9890-5 24736882

[46] Ciprandi G DeAmici M. Soluble HLA-G Serum Levels Depend on Allergy Type and IgE Levels. Allergy Rhinol (Providence) (2014) 5:9–11. doi: 10.2500/ar.2014.5.0076 24612937 PMC4019747

[47] Tahan F Gungor HE Akar HH Saraymen B. Increased Plasma Soluble Human Leukocyte Antigen-G in Persistent Wheezy Infants. Ped Int (2017) 59:530–3. doi: 10.1111/ped.13207 27880031

[48] Tahan F Patiroglu T. Plasma Soluble Human Leukocyte Antigen G Levels in Asthmatic Children. Int Arch Allergy Immunol (2006) 141:213–6. doi: 10.1159/000095290 16926540

[49] Zheng XQ Li CC Xu DP Lin A Bao WG Yang GS. Analysis of the Plasma Soluble Human Leukocyte Antigen-G and Interleukin-10 Levels in Childhood Atopic Asthma. Hum Immunol (2010) 71:982–7. doi: 10.1016/j.humimm.2010.06.018 20600443

[50] White SR Loisel DA McConville JF Stern R Tu Y Marroquin BA. Levels of Soluble Human Leukocyte Antigen-G Are Increased in Asthmatic Airways. Eur Resp J (2010) 35:925–7. doi: 10.1183/09031936.00164809 PMC4285585 20356990

[51] Murdaca G Contini P Negrini S Ciprandi G Puppo F. Immunoregulatory Role of HLA-G in Allergic Diseases. J Immunol Res (2016) 2016:6865758. doi: 10.1155/2016/6865758 27413762 PMC4931064

[52] Ciprandi G Contini P Fenoglio D Sormani MP Negrini S Puppo F. Relationship Between Soluble HLA-G and HLA-A,-B,-C Serum Levels, and Interferon-Gamma Production After Sublingual Immunotherapy in Patients With Allergic Rhinitis. Hum Immunol (2008) 69:409–13. doi: 10.1016/j.humimm.2008.05.009 18573288

[53] Ciprandi G Contini P Pistorio A Murdaca G Puppo F. Sublingual Immunotherapy Reduces Soluble HLA-G and HLA-A,-B,-C Serum Levels in Patients With Allergic Rhinitis. Int Immunopharmacol (2009) 9:253–7. doi: 10.1016/j.intimp.2008.11.009 19100344

[54] El-Shahaway AA Morad EA Abd-Elbary ME. Evaluation of Soluble HLA-G Serum Level as Diagnostic Biomarker in Allergic Rhinitis Patients and Its Association With Specific IgE Levels. Egypt J Immunol (2018) 25:125–32. 30600955

[55] Ciprandi G De Amici M Caimmi M Marseglia A Marchi A Castellazzi AM. Soluble Serum HLA-G in Children With Allergic Rhinitis and Asthma. J Biol Regul Homeost Agents (2010) 24:221–4. 20487636

[56] Ober C. HLA-G: An Asthma Gene on Chromosome 6p. Immunol Allergy Clin N Am (2005) 25:669–79. doi: 10.1016/j.iac.2005.08.001 16257632

[57] Nicolae D Cox NJ Lester LA Schneider D Tan Z Billstrand C. Fine Mapping and Positional Candidate Studies Identify HLA-G as an Asthma Susceptibility Gene on Chromosome 6p21. Am J Hum Genet (2005) 76:349–57. doi: 10.1086/427763 PMC1196380 15611928

[58] Granada M Wilk JB Tuzova M Strachan DP Weidinger S Albrecht E. A Genome-Wide Association Study of Plasma Total IgE Concentrations in the Framingham Heart Study. J Allergy Clin Immunol (2012) 129:840–5. doi: 10.1016/j.jaci.2011.09.029 PMC3293994 22075330

[59] Nicodemus-Johnson J Laxman B Stern RK Sudi J Tierney CN Norwick L. Maternal Asthma and microRNA Regulation of Soluble HLA-G in the Airway. J Allergy Clin Immunol (2013) 131:1496–503. doi: 10.1016/j.jaci.2013.01.037 PMC3779062 23534973

[60] Tan Z Randall G Fan J Camoretti-Mercado B Brockman-Schneider R Pan L. Allele-Specific Targeting of microRNAs to HLA-G and Risk of Asthma. Am J Hum Genet (2007) 81:829–34. doi: 10.1086/521200 PMC2227932 17847008

[61] Ribeyre C Carlini F René C. HLA-G Haplotypes Are Differentially Associated With Asthmatic Features. Front Immunol (2018) 9:278. doi: 10.3389/fimmu.2018.00278 29527207 PMC5829031

[62] Alves CC Arrudab LKP Oliveira FR Massaro JD Aquino BJ Paz MA. Human Leukocyte Antigen-G 3’ Untranslated Region Polymorphisms Are Associated With Asthma Severity. Mol Immunol (2018) 101:500–6. doi: 10.1016/j.molimm.2018.08.013 30142579

[63] White SR Nicodemus-Johnson J Laxman B Denner DR Naureckas ET Hogarth D. Elevated Levels of Soluble Human Leukocyte Antigen-G in the Airways Are a Marker for a Low-Inflammatory Endotype of Asthma. J Allergy Clin Immunol (2017) 140:857–60. doi: 10.1016/j.jaci.2017.02.031 PMC5591772 28363527

[64] Carlini F Picard C Garulli C Piquemal D Roubertoux P Chiaroni J. Bronchial Epithelial Cells From Asthmatic Patients Display Less Functional HLA-G Isoform Expression. Front Immunol (2017) 8:6. doi: 10.3389/fimmu.2017.00006 28303134 PMC5333864

[65] Rizzo R Stignani M Amoudruz P Nilsson C Melchiorri L Baricordi O. Allergic Women Have Reduced sHLA-G Plasma Levels at Delivery. Am J Reprod Immunol (2009) 61:368–76. doi: 10.1111/j.1600-0897.2009.00703.x 19341387

[66] Mapp CE Ferrazzoni S Rizzo R Miotto D Stignani M Boschetto P. Soluble Human Leucocyte Antigen-G and Interleukin-10 Levels in Isocyanate-Induced Asthma. Clin Exp Allergy (2009) 39:812–9. doi: 10.1111/j.1365-2222.2009.03215.x 19302248

[67] Le Page MEL Goodridge JP Zhang G Holt PG Sly P Witt CS. Genetic Polymorphism of KIR2DL4 (CD158d), a Putative NK Cell Receptor for HLA-G, Does Not Influence Susceptibility to Asthma. Tissue Antigens (2013) 82:276–9. doi: 10.1111/tan.12185 24033084

[68] Maeda N Yamada C Takahashi A Kuroki K Maenaka K. Therapeutic Application of Human Leukocyte Antigen-G1 Improves Atopic Dermatitis-Like Skin Lesions in Mice. Int Immunopharmacol (2017) 50:202–7. doi: 10.1016/j.intimp.2017.06.026 28675838

[69] Contini P Puppo F Canonica GW Murdaca G Ciprandi G. Allergen-Driven HLA-G Expression and Secretion in Peripheral Blood Mononuclear Cells From Allergic Rhinitis Patients. Hum Immunol (2016) 77:1172–8. doi: 10.1016/j.humimm.2016.08.005 27527921

[70] Sørensen AE Johnsen CR Dalgaard LT Würtzen PA Kristensen B Larsen MH. Human Leukocyte Antigen-G and Regulatory T Cells During Specific Immunotherapy for Pollen Allergy. Int Arch Allergy Immunol (2013) 162(3):237–52. doi: 10.1159/000353281 24022071

[71] Catamo E Zupin L Segat L Celsi F Crovella S. HLA-G and Susceptibility to Develop Celiac Disease. Hum Immunol (2015) 76:36–41. doi: 10.1016/j.humimm.2014.12.006 25500250

[72] Catamo E Addobbati C Segat L Sotero Fragoso T Domingues Barbosa A Tavares Dantas A. HLA-G Gene Polymorphisms Associated With Susceptibility to Rheumatoid Arthritis Disease and Its Severity in Brazilian Patients. Tissue Antigens (2014) 84:308–15. doi: 10.1111/tan.12396 24957665

[73] Zhang X Li S Zhang Y Lu Y Wang J Xu J. Meta-Analysis of the Relationship Between 14bp Insertion/Deletion Polymorphism of HLA-G Gene and Susceptibility to Systemic Lupus Erythematosus. Hum Immunol (2014) 75:1171–6. doi: 10.1016/j.humimm.2014.10.008 25454623

[74] Negrini S Contini P Pupo F Greco M Murdaca G Puppo F. Expression of Membrane-Bound Human Leucocyte Antigen-G in Systemic Sclerosis and Systemic Lupus Erythematosus. Hum Immunol (2020) 81:162–7. doi: 10.1016/j.humimm.2019.12.004 31848026

[75] Favoino E Favia IE Vettori S Vicenti C Prete M Valentini G. Clinical Correlates of Human Leucocyte Antigen (HLA)-G in Systemic Sclerosis. Clin Exp Immunol (2015) 181:100–9. doi: 10.1111/cei.12633 PMC4469159 25847615

[76] Contini P Negrini S Murdaca G Borro M Puppo F. Evaluation of Membrane-Bound and Soluble Forms of Human Leucocyte Antigen-G in Systemic Sclerosis. Clin Exp Immunol (2018) 193:152–9. doi: 10.1111/cei.13134 PMC6046504 29660112

[77] Mohammadi N Adib M Alsahebfosoul F Kazemi M M. Etemadifar M. An Investigation Into the Association Between HLA-G 14 Bp Insertion/Deletion Polymorphism and Multiple Sclerosis Susceptibility. J Neuroimmunol (2016) 290:115–8. doi: 10.1016/j.jneuroim.2015.11.019 26711580

[78] Fainardi E Bortolotti D Bolzani S Castellazzi M Tamborino C Roversi G. Cerebrospinal Fluid Amounts of HLA-G in Dimeric Form Are Strongly Associated to Patients With MRI Inactive Multiple Sclerosis. Multiple Sclerosis J (2016) 22:245–9. doi: 10.1177/1352458515590647 26084349

[79] Borghi A Rizzo R Corazza M Bertoldi AM Bortolotti D Sturabotti G. HLA-G 14-Bp Polymorphism: A Possible Marker of Systemic Treatment Response in Psoriasis Vulgaris? Preliminary Results of a Retrospective Study. Dermatol Ther (2014) 27:284–9. doi: 10.1111/dth.12140 24909182

[80] Han M Jiang Y Lao K Xu X Zhan S Wang Y. sHLA-G Involved in the Apoptosis of Decidual Natural Killer Cells Following Toxoplasma Gondii Infection. Inflammation (2014) 37:1718–27. doi: 10.1007/s10753-014-9900-7 24854161

[81] Sadissou I d’Almeida T Cottrell G Luty A Krawice-Radanne I Massougbodji A. High Plasma Levels of HLA-G Are Associated With Low Birth Weight and With an Increased Risk of Malaria in Infancy. Malaria J (2104) 13:312. doi: 10.1186/1475-2875-13-312 PMC4248443 25115633

[82] Oliveira Souza DMB Genre J Alves Silva TG Soares CP Rocha KB Oliveira CN. Upregulation of Soluble HLA-G5 and HLA-G6 Isoforms in the Milder Histopathological Stages of Helicobacter Pylori Infection: A Role for Subverting Immune Responses? Scand J Immunol (2016) 83:38–43. doi: 10.1111/sji.12385 26346688

[83] Laaribi AB Zidi I Hannachi N Ben Yahia H Chaouch H Bortolotti D. Association of an HLA- G 14-Bp Insertion/Deletion Polymorphism With High HBV Replication in Chronic Hepatitis. J Vir Hepat (2015) 22:835–41. doi: 10.1111/jvh.12395 25619305

[84] Murdaca G Contini P Cagnati P Marenco S Pieri G Lantieri D. Behavior of Soluble HLA-A, -B, -C and HLA-G Molecules in Patients With Chronic Hepatitis C Virus Infection Undergoing Pegylated Interferon-Alpha and Ribavirin Treatment: Potential Role as Markers of Response to Antiviral Therapy. Clin Exp Med (2017) 17:93–100. doi: 10.1007/s10238-015-0399-5 26567007

[85] Murdaca G Contini P Setti M Cagnati P Lantieri F Indiveri F. Behavior of Non-Classical Soluble HLA Class G Antigens in Human Immunodeficiency Virus 1-Infected Patients Before and After HAART: Comparison With Classical Soluble HLA-A, -B, -C Antigens and Potential Role in Immune-Reconstitution. Clin Immunol (2009) 133:238–44. doi: 10.1016/j.clim.2009.08.002 19762282

[86] Zheng J Xu C Chu D Zhang X Li J Ji G. Human Leukocyte Antigen G is Associated With Esophageal Squamous Cell Carcinoma Progression and Poor Prognosis. Immunol Lett (2104) 161:13–9. doi: 10.1016/j.imlet.2014.04.007 24768599

[87] Guo ZY Lv YG Wang L Shi SJ Yang F Zheng GX. Predictive Value of HLA- G and HLA-E in the Prognosis of Colorectal Cancer Patients. Cell Immunol (2015) 293:10–6. doi: 10.1016/j.cellimm.2014.10.003 25461612

[88] Xu DP Shi WW Zhang TT Lv HY Li JB Lin A. Elevation of HLA-G-Expressing DC-10 Cells in Patients With Gastric Cancer. Hum Immunol (2016) 77:800–4. doi: 10.1016/j.humimm.2016.01.003 26773190

[89] König L Kasimir-Bauer S Hoffmann O Bittner AK Wagner B Manvailer LF. The Prognostic Impact of Soluble and Vesicular HLA-G and Its Relationship to Circulating Tumor Cells in Neoadjuvant Treated Breast Cancer Patients. Hum Immunol (2016) 77:791–9. doi: 10.1016/j.humimm.2016.01.002 26796737

[90] Wisniewski A Kowal A Wyrodek E Nowak I Majorczyk E Wagner M. Genetic Polymorphisms and Expression of HLA-G and Its Receptors, KIR2DL4 and LILRB1, in Non-Small Cell Lung Cancer. Tissue Antigens (2015) 85:466–75. doi: 10.1111/tan.12561 25855135

[91] Murdaca G Calamaro P Lantieri F Pigozzi S Mastracci L Grillo F. HLA-G Expression in Gastric Carcinoma: Clinicopathological Correlations and Prognostic Impact. Virchows Arch (2018) 473:425–33. doi: 10.1007/s00428-018-2379-0 29845360

[92] Johansen LL Lock-Andersen J Hviid TV. The Pathophysiological Impact of HLA Class Ia and HLA-G Expression and Regulatory T Cells in Malignant Melanoma: A Review. J Immunol Res (2016) 2016:6829283. doi: 10.1155/2016/6829283 27999823 PMC5141560

